# CCL21 Facilitates Chemoresistance and Cancer Stem Cell-Like Properties of Colorectal Cancer Cells through AKT/GSK-3*β*/Snail Signals

**DOI:** 10.1155/2016/5874127

**Published:** 2015-12-28

**Authors:** Lin-Lin Lu, Xiao-Hui Chen, Ge Zhang, Zong-Cai Liu, Nong Wu, Hao Wang, Yi-Fei Qi, Hong-Sheng Wang, Shao Hui Cai, Jun Du

**Affiliations:** ^1^Department of Microbial and Biochemical Pharmacy, School of Pharmaceutical Sciences, Sun Yat-sen University, No. 132 Waihuandong Road, University Town, Guangzhou 510006, China; ^2^Department of Pharmacology, School of Pharmaceutical Sciences, Jinan University, Guangzhou 510632, China

## Abstract

Some evidence indicated that chemoresistance associates with the acquisition of cancer stem-like properties. Recent studies suggested that chemokines can promote the chemoresistance and stem cell properties in various cancer cells, while the underling mechanism is still not completely illustrated. In our study, we found that CCL21 can upregulate the expression of P-glycoprotein (P-gp) and stem cell property markers such as Bmi-1, Nanog, and OCT-4 in colorectal cancer (CRC) HCT116 cells and then improve the cell survival rate and mammosphere formation. Our results suggested that Snail was crucial for CCL21-mediated chemoresistance and cancer stem cell property in CRC cells. Further, we observed that CCL21 treatment increased the protein but not mRNA levels of Snail, which suggested that CCL21 upregulates Snail via posttranscriptional ways. The downstream signals AKT/GSK-3*β* mediated CCL21 induced the upregulation of Snail due to the fact that CCL21 treatment can obviously phosphorylate both AKT and GSK-3*β*. The inhibitor of PI3K/Akt, LY294002 significantly abolished CCL21 induced chemoresistance and mammosphere formation of HCT116 cells. Collectively, our results in the present study revealed that CCL21 can facilitate chemoresistance and stem cell property of CRC cells via the upregulation of P-gp, Bmi-1, Nanog, and OCT-4 through AKT/GSK-3*β*/Snail signals, which suggested a potential therapeutic approach to CRC patients.

## 1. Introduction

CCL21 belongs to chemoattractant cytokines family. Chemokines are small proteins that weighed up to 8–10 kDa, which are divided into four types on the basis of the location of the first two cysteine residues: C, CC, CXC and CX_3_C [1]. Chemokine receptors are G protein-coupled receptors (GPCRs) that trigger intracellular signaling pathways via conformational changes, which can regulate the activation and movement of leukocytes. CCL21, CCL19, and their receptor CC chemokines receptor 7 (CCR7) play an important role in directing T cells and DCs to lymph organs, which make a contribution to immunity and tolerance. CCR7 has been detected in various immune cells and promotes cell migration toward CCL19 and CCL21, which are considered as inflammatory cytokines due to the fact that they appear in most chronically inflamed organs [2]. CCR7/CCL19/CCL21 axis can establish and propagate anatomical microenvironment, which helps the relations between antigen presenting cells and antigen specific lymphocytes and plays an important role in effective adaptive immune system function [3]. CCL19, CCL21, and their receptor have multiple roles including lymphocyte egress from tissues, high similar antibody responses, secondary lymphoid organogenesis, and memory and regulatory T cell role [3]. Recent studies revealed that chemokines such as IL-8 can strength the chemoresistance of cancer cells [4] and cancer stem cell-like properties [5], indicating that chemokines may be related to these process. But there is no study reporting that CCL21 can promote chemoresistance and cancer stem cell properties and the underling mechanism is not unclear.

Cancer stem cells (CSCs), firstly presented in 1997, when only CD34+CD38− gave rise to patients with acute myeloid leukemia (AML) in nonobese diabetic/severe combined with immunodeficient mice, could induce hematopoietic malignancies demonstrated by Bonnet and Dick [6]. Since then, a number of solid tumors cancer stem cells have been identified, for instance, melanoma [7], breast cancer [8], lung cancer [9], colon cancer [10], and brain tumors [11]. In neoplastic cells, CSCs constitute a small minority that they have the ability to self-renew and to use the common signaling pathways with their progeny, which are different from them. Cancer stem cells may make a contribution to the source of tumor cells in malignant tumors and differentiation into multiple cell types. Some evidence indicated that pluripotent embryonic stem-like cells can be reprogrammed from the somatic cells with coexpression of pluripotent markers such as Sox2, Lin28, Bmi-1, Oct-4, and Nanog [12]. Cancer stem cells performed resistance to chemotherapeutic agents, which were the source of cells that cause distant metastasis [13], bringing much trouble to tumor therapy. However, the molecular mechanisms of how CSCs involve in drug resistance need to be explained. A recent study showed that drug-resistant cancer cells perform stem-like feature [12]. Therefore, these findings indicated that cancer stem cell-like properties are closely associated with chemoresistance.

Chemoresistance is closely related to multidrug resistance (MDR). P-gp is a 170 kD membrane protein encoded by MDR1 gene, also known as ABCB1 [14]. This protein was first found in drug-resistant cells, also played a vital role in transporting endogenous metabolites and xenobiotics, and is active transporter. MDR1 has much role including regulating the cellular absorption and the toxicity of pharmacological agents and the metabolism [15, 16]. So, to a large extent, MDR1 could impact the efficiency of agents and effects of the treatment. Even a single nucleotide polymorphisms in ABC drug-efflux pumps could influence the drug and disease susceptibility [17–19].

In the present study, we examined whether CCL21 in HCT116 cells could facilitate chemoresistance and signatures of CSCs such as mammosphere forming ability. We demonstrated that activation of CCR7 by CCL21 can induce increased cell viability and mammosphere forming ability of colorectal cells via AKT/GSK-3*β* signals. Further, the CCL21 treatment significantly upregulated the expression of P-gp, Bmi-1, Nanog, and Oct-4 of HCT116 cells and increased the survival rate and mammosphere forming rate. Our study suggested that CCL21 might lay the foundation for future development of CCL21-based therapies for colorectal cancer treatment.

## 2. Materials and Methods

### 2.1. Chemicals and Reagents

*噻唑蓝* (MTT), Rhodamine 123 (Rh123), MAPK inhibitor PD98059, TGF-*β*/Smad2 inhibitor SB431542, p38MAPK inhibitor SB203580, JAK/Stat3 inhibitor AG490, PI3K inhibitor LY294002, and proteasome inhibitor MG132 were bought from Sigma-Aldrich (St. Louis, MO). Primary antibodies against P-gp, Snail, AKT, p-AKT (Ser473), p38, p-p38 (Thr180/Tyr182), Smad2, p-Smad2 (Ser465/467), Stat3, p-Stat3 (Tyr705), GSK-3*β*, p-GSK-3*β* (Ser9), and *β*-catenin were bought from Cell Signaling Technology (MA, USA). Recombinant human CCL21 protein was obtained from Peprotech. PrimeScript RT reagent Kit and SYBR Premix Ex Taq TM were purchased from TaKaRa.E.Z.N.AR HP Total RNA Kit, the product of Omega Bio-Tek (Doraville, USA). Smart pool siRNA against human snail (si-Snail) and control (SiNC) were gained from RiboBio (Guangzhou, China). Doxorubicin (Dox), 5-fluorouracil (5-FU) were purchased from Zhejiang HISUN Pharmaceuticals Co. (Zhejiang, China). Vectors (pGL3-Basic and pRL-TK) and dual-luciferase assay kit were products of Promega (Madison, WI, USA). PGL3-Snail-luc reporter gene plasmid was previously constructed and examined in our laboratory [20].

### 2.2. Cell Lines and Culture

HCT116 cell lines were purchased from the Culture Collection of the Chinese Academy of Sciences (Shanghai, China). Cell were cultured in RPMI 1640 medium (Gibco BRL) supplemented with 10% fetal bovine serum under a humidified 5% CO_2_ atmosphere at 37°C in incubator.

### 2.3. Cell Viability Assay

Cells were seeded in 96-well plates at 1 × 10^4^ cells per well in 10% FBS-supplemented 1640. The following day, the cells were treated with 0.15, 0.5, 1.5, 5, 15, and 50 *μ*M Dox and 5-FU for 48 h. Then, cell growth was evaluated using an MTT cell viability assay system according to the manufacturer's protocol. Following the colorimetric reaction, the optical density was determined at 490 nm using Enzyme Standard Instrument (BioRad, USA) [21].

### 2.4. Mammosphere Formation Assay

Mammosphere formation assay dependent on the capability that stem/progenitor cells have to grow and form spheres in serum-free medium. Mammosphere culture was done in a serum-free DMEM/F12 (Invitrogen) supplemented with B27 (Invitrogen), 20 ng/mL EGF (Invitrogen), 20 ng/mL basic fibroblast growth factor (bFGF) (Invitrogen), 5 g/mL insulin, 1 g/mL hydrocortisone, and 1% antibiotic-antimycotic. Single cells prepared from mechanical and enzymatic dissociation were plated in 24-well ultralow attachment plates (Corning) at a density of 1000 cells/mL in culture. Single cell status was confirmed under microscope. Fresh mammosphere culture was added every 3-4 days. After 7 days of culture, the number of mammospheres (>20 *μ*m) was counted under an upright microscope and the photos were acquired with the upright microscope. Each assay was carried out in triplicate and repeated in three independent experiments.

### 2.5. Quantitative Real-Time PCR

Total mRNA of the cells was extracted after treatment for the indicated time. First strand cDNA synthesis was generated from 500 ng of total RNA. Quantification of target and reference (GAPDH) genes was performed in triplicate on LightCycler 480 II (Roche, Applied Science). The primers used in each reaction were as follows: Snail, forward 5′- GAC CAC TAT GCC GCG CTC TT-3′ and reverse 5′-TCG CTG TAG TTA GGC TTC CGA TT-3′. MDR1, forward 5′- TCG TTT CCT TTA GGT CTT TCC AC-3′ and reverse 5′-CTT CTT CTT TGC TCC TCC ATT GC-3′. GAPDH, forward 5′-GCA CCG TCA AGG CTG AGA AC-3′ and reverse 5′-TGG TGA AGA CGC CAG TGG A-3′. After being normalized to GAPDH gene, expression levels for each target gene were calculated using the comparative threshold cycle (CT) method. The Dct values were calculated according to the formula Δct = ct (gene of interest) − ct (GAPDH) in correlation analysis, and 2^−ΔΔct^ was calculated according to the formula ΔΔct = Δct (control group) −  Δct (experimental group) for determination of relative.

### 2.6. Western Blotting Analysis

The cells were washed three times with ice-cold phosphate buffer solution (PBS) and then lysed in lysis buffer containing 50 mM Tris-HCl (pH 7.6), 150 mM NaCl, 1 mM EDTA, 1% NP-40, 0.5% Na-deoxycholate, 5 mg/mL aprotinin, 5 mg/mL leupeptin, and 1 mM phenylmethylsulfonyl fluoride. Lysates were cleared by centrifugation and denatured by boiling in Laemmli buffer. Equal amounts of protein samples were loaded per well and separated on SDS-polyacrylamide gels and then electrophoretically transferred onto PVDF membranes. Following blocking with 5% nonfat milk at room temperature for 2 h, membranes were incubated with primary antibodies (1 : 1,000 dilution) at 4°C overnight and then incubated with HRP-conjugated secondary antibodies (1 : 5,000 dilution) for 2 h at room temperature. Specific immune complexes were detected using Western Blotting Plus Chemiluminescence Reagent (Life Science).

### 2.7. Gene Overexpression and RNA Interference

Cells were seeded on a 6-well plate (2 × 10^5^ cells/well) and cultured for 24 h. They were then transfected with 2 *μ*g plasmid vector or 100 pmol siRNA mixed with Lipofectamine 2000 reagent in serum reduced medium according to the manufacturer's instructions. Medium was changed to complete culture medium 4 h later, and the cells were incubated at 37°C in a CO_2_ incubator for another 24 h before harvest.

### 2.8. Analysis of Fluorescence Intensity by Flow Cytometry

For visualization of the effects of CCL21 on the intracellular retention of Rh123, 5 × 10^5^ cells were seeded on 6-well plate slides on the day prior to the assay and treatment with CCL21 (200 ng) for 72 h at 37°C. Then, cells were incubated with either 5 mM Rh123 alone at 37°C for 2 h [22]. After the cells were washed for three times with cold PBS, images were acquired by fluorescence microscopy (Olympus, Japan) 488 nm excitation and 535 nm emission wavelength [23, 24].

### 2.9. Statistical Analysis

Results were expressed as mean ± standard deviation (SD) of three independent experiments unless otherwise specified. Data were analyzed by two-tailed unpaired Student's *t*-test between any two groups. These analyses were performed using GraphPad Prism Software Version 5.0 (GraphPad Software Inc., La Jolla, CA). FlowJo software A (Ashland, OR) was used to analyze the data of flow cytometry. *P* value of <0.05 was considered as statistically significant.

## 3. Results

### 3.1. CCL21 Promotes Chemoresistance and Upregulates P-gp in HCT116 Cells

**Figure 1:**
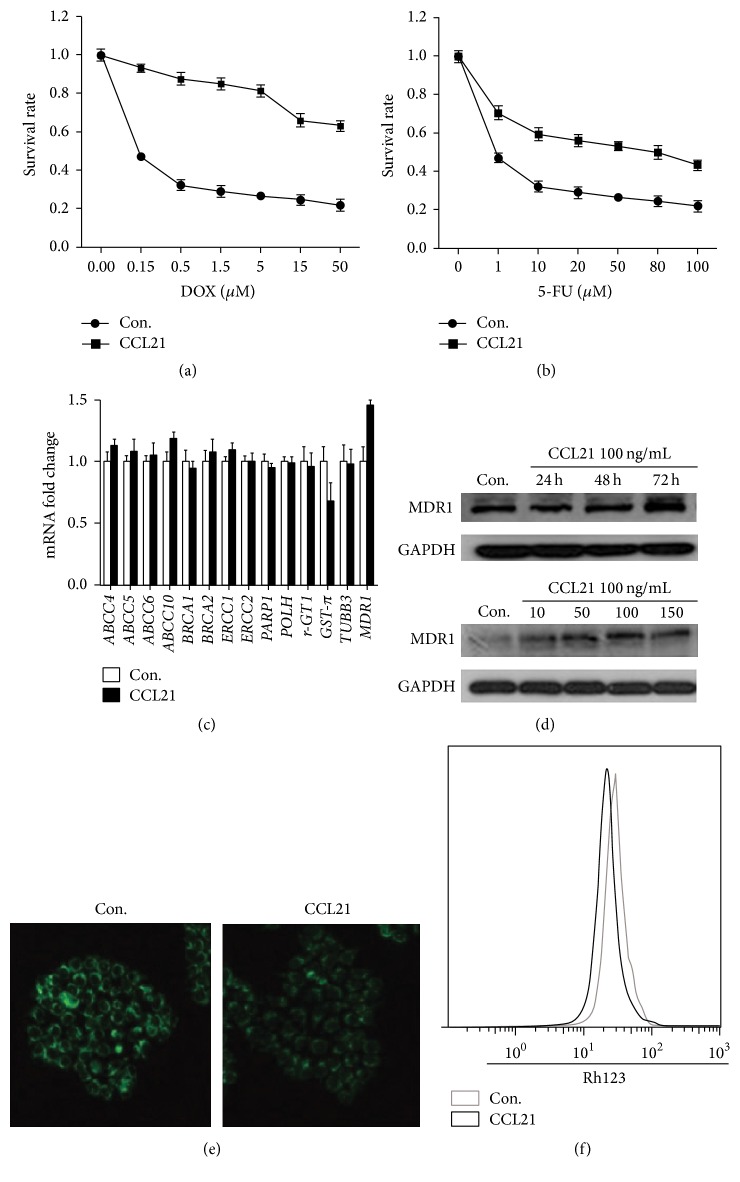
CCL21 promotes chemoresistance in HCT116 cells. (a) HCT116 cells were treated with or without CCL21 (100 ng/mL) for 72 h and then were exposed to doxorubicin (DOX) for 48 h and measured the cell viability by MTT. (b) HCT116 cells were treated with or without CCL21 (100 ng/mL) for 72 h and were then exposed to 5-FU for 48 h and measured the cell viability by MTT. (c) HCT116 cells were treated with or without CCL21 (100 ng/mL) for 24 h. The mRNA levels of several chemoresistance genes were analyzed by qRT-PCR. ^*∗*^ *P* < 0.05. (d) HCT116 cells were treated with or without CCL21 (100 ng/mL) for 24 h, 48 h, and 72 h, and the protein level of P-gp was analyzed by western blotting. HCT116 cells were treated with CCL21 (10, 50, 80, 100, and 150 ng/mL) for 72 h, and the protein level of P-gp was analyzed by western blotting. (e) HCT116 cells were treated with CCL21 (100 ng/mL) for 72 h, and then Rh123 (5 *μ*M) was added to the cells and incubated for 2 h. The cells were washed five times with cold PBS and fluorescent intensity images were obtained via fluorescent inverted microscope. (f) HCT116 cells were treated with CCL21 (100 ng/mL) for 72 h, and then Rh123 (5 *μ*M) was added to the cells and incubated for 2 h. The cells were washed five times with cold PBS and digested and we used the flow cytometry to detect fluorescent intensity.

The IC_50_ of HCT116 cells influenced by CCL21 were measured by MTT. The DOX IC_50_ of HCT116 cells treated with or without CCL21 are 70 *μ*M and 0.15 *μ*M, respectively (Figure 1(a)). The 5-FU IC_50_ of HCT116 cells treated with or without CCL21 are 50 *μ*M and 0.15 *μ*M, respectively (Figure 1(b)). To identify genes that make a contribution to CCL21 induced chemoresistance, we analyzed the chemoresistance related genes by real-time RT-PCR in HCT116 cells treated with or without CCL21. Our results showed that only MDR1, the P-gp gene, had a dramatic increase in HCT116 cells when treated with CCL21 (Figure 1(c)). Further, CCL21 treatment can increase the protein levels of P-gp via both time and dose dependent manners (Figure 1(d)). That CCL21 upregulating P-gp in HCT116 cells was also confirmed by the results of Rh123 tests. The fluorescent intensity for cells treated with CCL21 was significantly less than that of control groups (Figure 1(e)), which was confirmed by the quantification analysis by the use of flow cytometry (Figure 1(f)). Collectively, our results revealed that CCL21 can promote chemoresistance and upregulation of P-gp in HCT116 cells.

### 3.2. CCL21 Induces Cancer Stem Cell-Like Properties of HCT116 Cells

**Figure 2:**
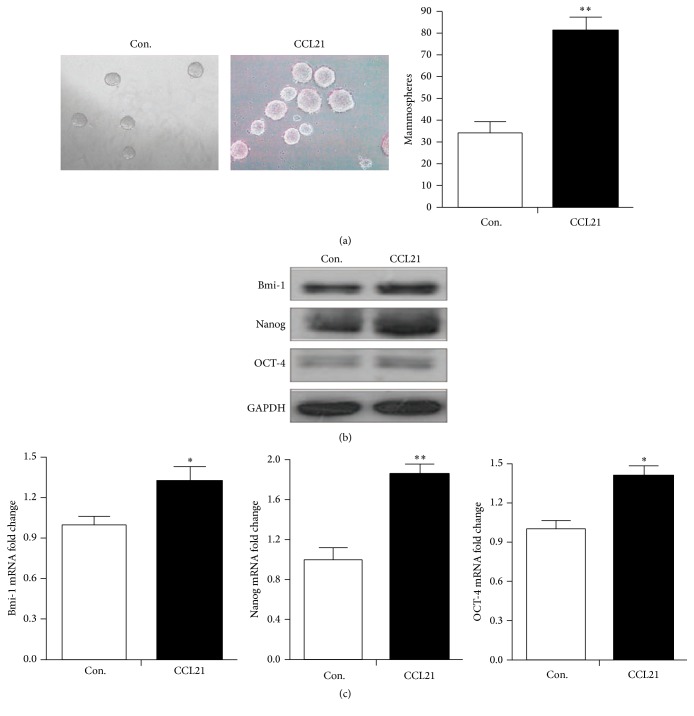
CCL21 promoted mammosphere forming in HCT116 cells. (a) HCT116 cells had enhanced mammosphere forming ability when treated with CCL21 (100 ng/mL). (b) HCT116 cells were treated with or without CCL21 (100 ng/mL) for 24 h, 48 h, and 72 h, and the protein levels of Bmi-1, Nanog, and Oct-4 were analyzed by western blotting. (c) HCT116 cells were treated with or without CCL21 (100 ng/mL) for 24 h. The mRNA levels of the above genes were analyzed by qRT-PCR. ^*∗*^ *P* < 0.05.

Because exposure to CCL21 could upregulate CSC markers, we investigated the effects of CCL21 on stem cell-like mammosphere forming phenotype in HCT116 cells. As shown in (Figure 2(a)), cells that were treated with recombinant human CCL21 exhibited an enhanced capacity of mammosphere forming compared to controls. The number of mammospheres of HCT116 cells treated with CCL21 increased about 2-fold compared to controls. To further identify the genes that maintain and regulate the stem cell properties in HCT116 cells, we analyzed the selected genes performing western blotting and real-time RT-PCR. Western blotting results showed that pluripotent markers Bmi-1, Nanog, and Oct-4 were significantly increased in cells treated with CCL21 compared to controls (Figure 2(b)). Furthermore, real-time RT-PCR analysis demonstrated that the expressions of Bmi-1, Nanog, and Oct-4 at mRNA level were also higher in HCT116 cells exposed to CCL21 compared to controls (Figure 2(c)). Collectively, our results revealed that CCL21 can induce cancer stem cell properties and upregulation of Bmi-1, Nanog, and Oct-4 in HCT116 cells.

### 3.3. Snail Is Crucial for CCL21-Mediated Chemoresistance and Cancer Stem Cell Properties of HCT116 Cells

**Figure 3:**
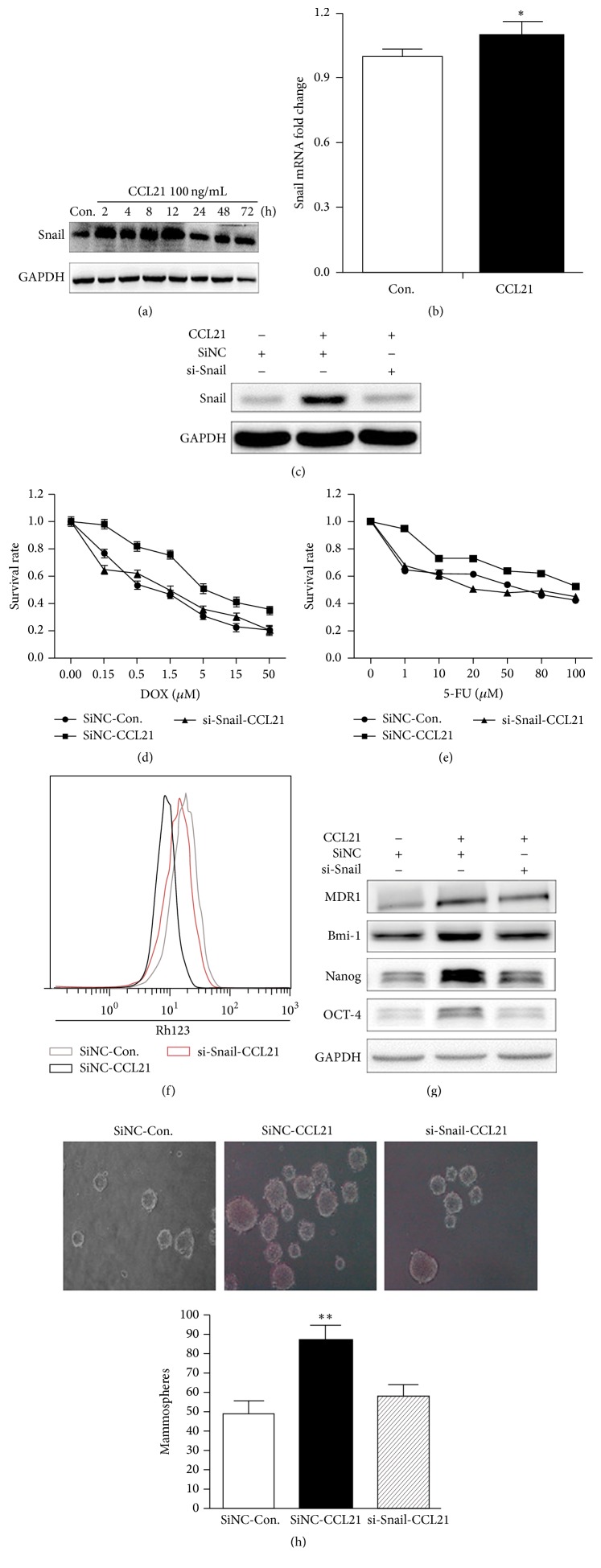
Snail is vital to chemoresistance and cancer stem cell properties promoted by CCL21. (a) HCT116 cells were treated with CCL21 (100 ng/mL) for 2, 4, 8, 12, 24, 48, and 72 h and the expression of Snail at protein level was analyzed by western blotting. (b) HCT116 cells were treated with CCL21 (100 ng/mL) for 24 h, and the expression of Snail at mRNA level was analysed by qRT-PCR. (c) SiNC or si-Snail siRNAs were transfected into cells for 24 h and treated with or without CCL21 (100 ng/mL) for 24 h, and then the expressions of Snail were detected by western blotting. (d) SiNC or si-Snail siRNAs were transfected into cells for 24 h and treated with or without CCL21 (100 ng/mL) for 72 h, and then the cell sensitivity to DOX was detected by MTT. (e) SiNC or si-Snail siRNAs were transfected into cells for 24 h and treated with or without CCL21 (100 ng/mL) for 72 h, and then the cell sensitivity to 5-FU was detected by MTT. (f) SiNC or si-Snail siRNAs were transfected into cells for 24 h and treated with or without CCL21 (100 ng/mL) for 72 h, and then RH123 (5 *μ*M) was added to cells for 2 h. Following that, the fluorescent intensity was analyzed via flow cytometry. (g) SiNC or si-Snail siRNAs were transfected into cells for 24 h and treated with or without CCL21 (100 ng/mL) for 72 h, and then the expressions of P-gp, Bmi-1, Nanog, and OCT-4 were detected by western blotting. (h) SiNC or si-Snail siRNAs were transfected into cells for 24 h and treated with or without CCL21 (100 ng/mL) for a week, detecting the mammosphere forming ability.

Recent studies revealed that Snail played an important role in chemoresistance [25], further, it was also reported that chemokines such as CXCL13 treatment can upregulate the expression of Snail in cancer cells and induce EMT [26]. Therefore, the mRNA and protein levels of Snail in HCT116 cells were measured by real-time PCR and western blotting, respectively. The results revealed that CCL21 can increase the protein levels of Snail via a time dependent manner (Figure 3(a)), while having limited effects on the mRNA expression (Figure 3(b)).

To verify the roles of Snail in CCL21 induced chemoresistance and stem cell properties, we knocked down Snail by its specific siRNA (Figure 3(c)) and then investigated the chemoresistance variation and mammosphere forming ability of HCT116 cells treated with or without CCL21. Our results showed that si-Snail significantly attenuated the CCL21 induced resistance to Dox (Figure 3(d)) and 5-FU (Figure 3(e)) and the amount of mammosphere (Figure 3(h)). Further, si-Snail also abolished CCL21 induced upregulation of P-gp, Bmi-1, Nanog, and OCT-4 (Figure 3(g)) and excretion of Rh123 (Figure 3(f)). These results suggested that Snail is crucial for CCL21-mediated chemoresistance and cancer stem cell properties in HCT116 cells.

### 3.4. Overexpression of Snail Induces Chemoresistance of HCT116 Cells

**Figure 4:**
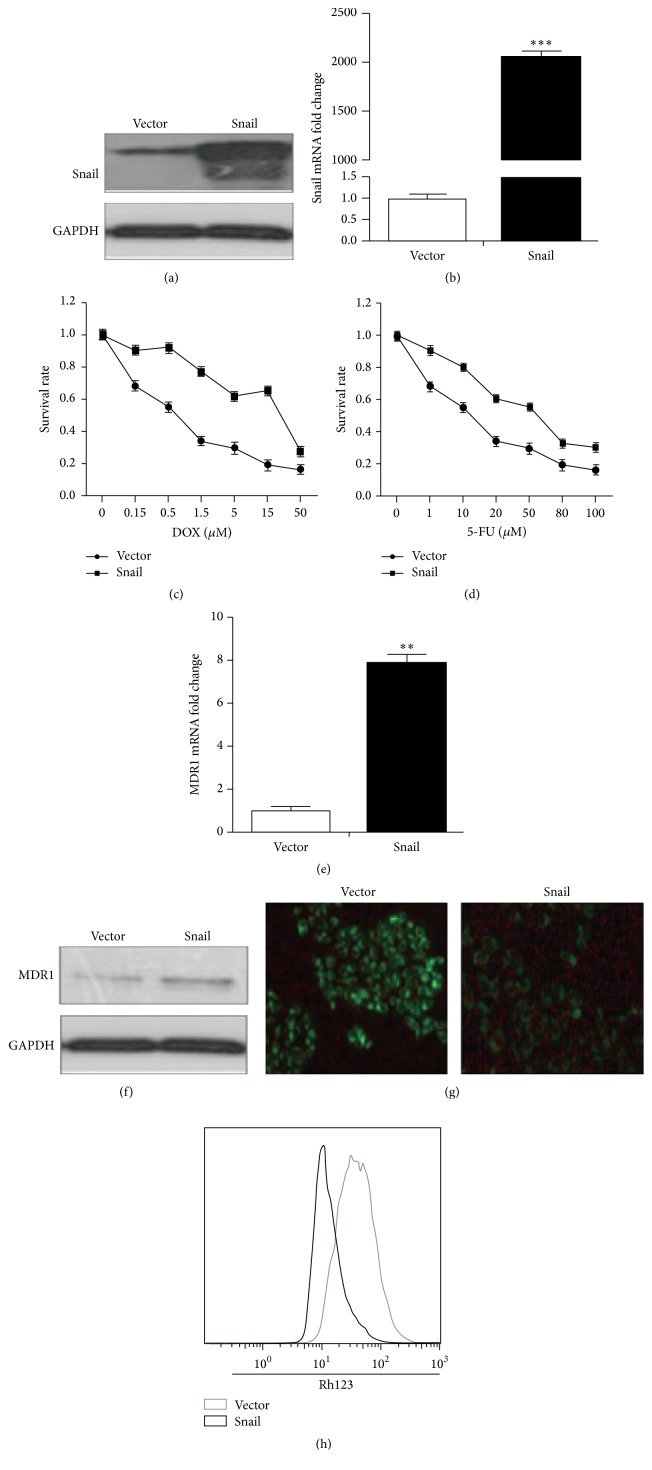
Snail is crucial for the CCL21 promoted chemoresistance. (a) HCT116 cells were transfected with control vector pcDNA-3.1 (Vector) and pcDNA-Snail for 24 h, and the expression of Snail at protein level was detected by western blotting. (b) HCT116 cells were transfected with control vector pcDNA-3.1 (Vector) and pcDNA-Snail for 24 h, and the expression of Snail at mRNA level was detected by qRT-PCR. (c) Control vector pcDNA-3.1 (Vector) and pcDNA-Snail were transfected into HCT116 cells for 48 h; the cell sensitivity to DOX was detected by MTT. (d) Control vector pcDNA-3.1 (Vector) and pcDNA-Snail were transfected into HCT116 cells for 48 h, and the cell sensitivity to 5-FU was detected by MTT. (e) Control vector pcDNA-3.1 (Vector) and pcDNA-Snail were transfected into HCT116 cells for 48 h, and the expression of P-gp at mRNA level was analyzed via qRT-PCR. (f) Control vector pcDNA-3.1 (Vector) and pcDNA-Snail were transfected into HCT116 cells for 48 h, and the expression of P-gp at protein level was analyzed via western blotting. (g) Control vector pcDNA-3.1 (Vector) and pcDNA-Snail were transfected into HCT116 cells for 48 h, and then RH123 (5 *μ*M) was added to the cells for 2 h. The cells were washed five times with cold PBS, and pictures were acquired by fluorescence microscopy. (h) Control vector pcDNA-3.1 (Vector) and pcDNA-Snail were transfected into HCT116 cells for 48 h, and then Rh123 (5 *μ*M) was added to the cells and incubated for 2 h. The cells were washed five times with cold PBS and digested, and we used the flow cytometry to detect fluorescent intensity.

**Figure 5:**
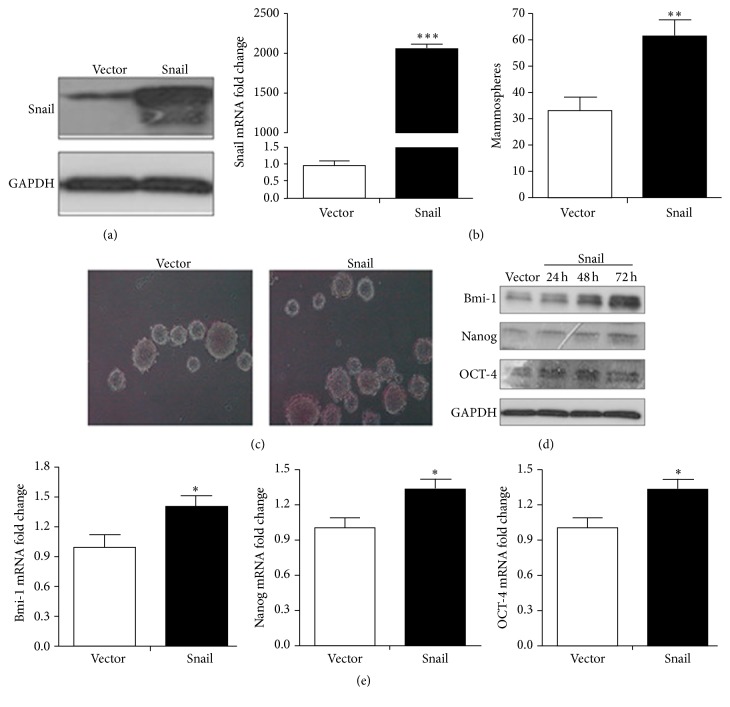
Snail is crucial for the CCL21 promoted cancer stem cell properties. (a) HCT116 cells were transfected with control vector pcDNA-3.1 (Vector) and pcDNA-Snail for 24 h, and the expression of Snail at protein level was detected by western blotting. (b) HCT116 cells were transfected with control vector pcDNA-3.1 (Vector) and pcDNA-Snail for 24 h, and the expression of Snail at mRNA level was detected by qRT-PCR. (c) Control vector pcDNA-3.1 (Vector) and pcDNA-Snail were transfected into HCT116 cells and were detecting the mammosphere forming ability. (d) Control vector pcDNA-3.1 (Vector) and pcDNA-Snail were transfected into HCT116 cells for 24 h, 48 h, and 72 h, and then the expressions of Bmi-1, Nanog, and Oct-4 at protein level were analyzed via western blotting. (e) Control vector pcDNA-3.1 (Vector) and pcDNA-Snail were transfected into HCT116 cells for 24 h, 48 h, and 72 h, and then the expressions of Bmi-1, Nanog, and Oct-4 at mRNA level were analyzed via qRT-PCR.

To further verify the role of Snail in CCL21 promoted chemoresistance, we overexpressed Snail in HCT116 cells by transfection of pcDNA3.1/Snail. The overexpression of Snail in HCT116 was confirmed by western blot (Figure 4(a)) and real-time PCR (Figure 4(b)). The cell viability assay showed that overexpression of Snail significantly reduced the sensitivity of Dox (Figure 4(c)) and 5-FU (Figure 4(d)) of HCT116 cells. Further, the overexpression of Snail also increased the mRNA (Figure 4(e)) and protein (Figure 4(f)) levels of P-gp in HCT116 cells. The Rh123 assays also indicated that overexpression of Snail can decrease the drug excretion in HCT116 cells, which was confirmed by both fluorescent intensity assay (Figure 4(g)) and flow cytometry (Figure 4(h)). These data indicated that the overexpression of Snail can induce chemoresistance in HCT116 cells.

### 3.5. Overexpression of Snail Promotes Stem Cell Properties of HCT116 Cells

To further identify the role Snail played in stem cell properties, we performed overexpressed Snail in HCT116 cells, and then the cells were transfected with pCDNA-3.1 or pCDNA-Snail. Firstly, the overexpression of Snail was detected using western blotting (Figure 5(a)) and quantitative real-time PCR (Figure 5(b)). The mammosphere forming ability assay indicated that the overexpression of Snail significantly increase the mammosphere (Figure 5(c)). Further, the overexpression of Snail increased the protein (Figure 5(d)) and mRNA (Figure 5(e)) levels of Bmi-1, Nanog, and Oct-4 in HCT116 cells. Our data suggested that the overexpression of Snail can induce cancer stem cell properties in HCT116 cells.

### 3.6. CCL21 Upregulates Snail via AKT/GSK-3*β* Signals in HCT116 Cells

**Figure 6:**
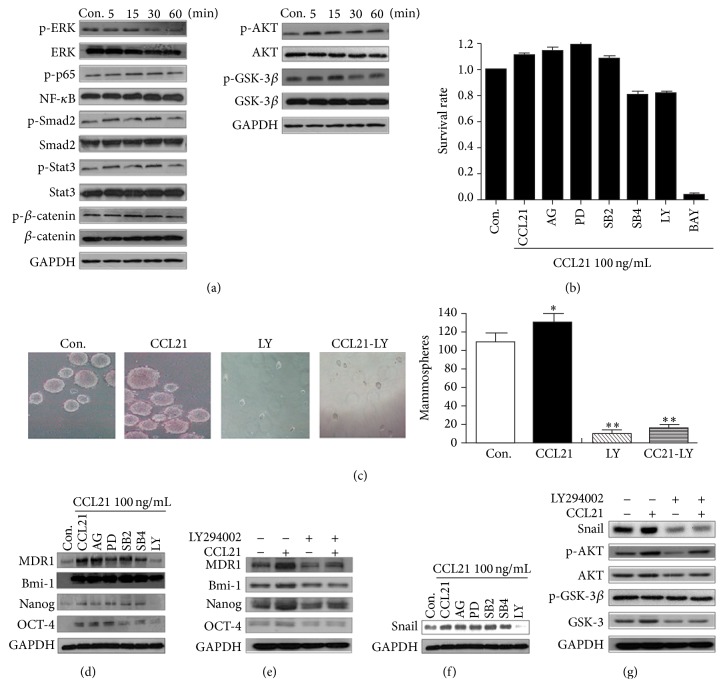
AKT/GSK-3*β* signaling regulates upregulated P-gp and stability of Snail in HCT116 cells. (a) HCT116 cells were treated with CCL21 for 5 min, 15 min, 30 min, and 1 h, and then several signaling pathway key proteins were detected via western blotting. (b) HCT116 cells were pretreated with or without AG490 (20*μ*M), SB431542 (20 *μ*M), SB203580 (20 *μ*M), PD98059 (20 *μ*M), BAY (10 *μ*M), or LY294002 (20 *μ*M) for 2 h, respectively, and treated with CCL21 (100 ng/mL) for 72 h, and then the cell viability was analyzed by MTT. (c) HCT116 cells were pretreated with or without LY294002 (20 *μ*M) for 2 h, respectively, and treated with CCL21 (100 ng/mL), detecting the mammosphere forming ability. (d) HCT116 cells were pretreated with or without AG490 (20 *μ*M), SB431542 (20 *μ*M), SB203580 (20 *μ*M), PD98059 (20 *μ*M), or LY294002 (20 *μ*M) for 2 h, respectively, and treated with CCL21 (100 ng/mL) for 72 h, and then the expressions of P-gp, Bmi-1, Nanog, and OCT-4 were analyzed via western blotting. (e) HCT116 cells were pretreated with or without LY294002 (20 *μ*M) for 2 h and then treated with or without CCL21 (100 ng/mL) for 72 h, and P-gp, Bmi-1, Nanog, and OCT-4 key protein were analyzed by western blotting. (f) HCT116 cells were pretreated with or without AG490 (20 *μ*M), SB431542 (20 *μ*M), SB203580 (20 *μ*M), PD98059 (20 *μ*M), or LY294002 (20 *μ*M) for 2 h, respectively, and treated with CCL21 (100 ng/mL) for 24 h, and then the expression of Snail was analyzed via western blotting. (g) HCT116 cells were pretreated with or without LY294002 (20 *μ*M) for 2 h and then treated with or without CCL21 (100 ng/mL) for 5 min, and Snail, AKT, and GSK-3*β* pathway key protein were analyzed by western blotting.

AKT pathway can be activated in various cancers, which is frequently involved in regulating Snail and makes a contribution to induce EMT [27]. To investigate whether AKT and other related signals were involved in CCL21 induced chemoresistance and cancer of HCT116 cells, the total and phosphorylation levels of AKT, NF-kappaB, Smad-2, Stat3, *β*-catenin, and GSK-3*β* were measured by western blotting. The results revealed that CCL21 significantly phosphorylated AKT and GSK-3*β* but not other molecules in HCT116 cells (Figure 6(a)). To test the roles of AKT in CCL21 induced chemoresistance and upregulation of P-gp in HCT116 cells, we pretreated cells with various inhibitors including PD98059, SB431542, SB203580, AG490, LY294002, or BAY, and then we treated the cells with CCL21. Our results revealed that only LY294002 inhibited both chemoresistance (Figure 6(b)) and P-gp upregulation (Figure 6(d)) in HCT116 cells. Furthermore, LY 294002 also abolished CCL21 induced mammosphere forming (Figure 6(c)) and upregulation of Bmi-1, Nanog, and OCT-4 (Figure 6(d)). Consistent with our suppose, LY 294002 also reversed CCL21 induced Snail upregulation (Figure 6(f)) and GSK-3*β* phosphorylation (Figure 6(g)). Considering that AKT/GSK-3*β* can upregulate the stabilization of Snail in HCT116 cells in our previous study [27], our results revealed that CCL21 upregulated Snail and promoted chemoresistance and stem cell properties via AKT/GSK-3*β* signals in HCT116 cells.

## 4. Discussion

Colon tumor now is one of the most common malignant cancers in gastrointestinal track, and the incidence of this tumor has obviously raised in China in the recent years [28]. CRC is widely treated with 5-fluorouracil (5-FU), but the resistance to this drug is a hard problem in cancer chemotherapy [29]. Changes in the expression of apoptosis-regulating genes make a contribution to the resistance. At present, MDR is a phenotype where cancer cells become resistant to a wide range of chemotherapeutics [30], which is a major challenge during tumor therapy, but the underlying mechanism is not fully understood. Some studies have shown that the emergence of MDR is related to the overexpression of P-gp [31], while the mechanisms responsible for P-gp upregulation in CRC cells remain to be illustrated.

CCL21 has a wide range of biological roles, which is an inflammatory cytokine. CCL21, CCL19, and its receptor CCR7 take part in a series of immunological processes including T cell homeostasis [32], the generation of thymocytes [33, 34], regulatory T cell (Treg) function [35–37], and central and peripheral tolerance [38, 39]. There has been study reporting that chemokines can promote chemoresistance [4] and cancer stem cell properties [40]. But there is no report about the role of CCL21 in this process. In our present study, the results showed that CCL21 promoted chemoresistance with upregulation of P-gp and stem cell properties with increased expression of Bmi-1, Nanog, and Oct-4 in HCT116 cells. CCL21 significantly increased the IC_50_ of DOX and 5-FU and mammosphere forming ability. To our knowledge, this is the first study to reveal that CCL21 is able to promote chemoresistance and cancer stem cell properties in HCT116 cells.

Several transcription factors have been involved in the control of chemoresistance and cancer stem cell properties, in which Snail, a zinc-finger transcription factor [25], has been known as a vital player in these process. Snail was significantly upregulated and was critical in the CCL21 promoted chemoresistance and stem cell properties in HCT116 cells. Basic fibroblast growth factor (bFGF) can also upregulate Snail in normal tissue cells and colon cancer cells such as HT29 and DLD-1 cells, which was able to induce EMT [41, 42]. To some extent, EMT has many relations with chemoresistance and cancer stem cell properties. Some recent studies reported that drug-resistant cancer cells acquire cancer stem cell-like properties and EMT features [43–45]. But no study has indicated the role of Snail in CCL21 promoted chemoresistance and cancer stem cell property in HCT116 cells. In our study, the results show that Snail played a vital role in CCL21 promoted chemoresistance and cancer stem cell properties. It was found that the expression of Snail exhibited an increase after CCL21 stimulation. Similarly, overexpression of Snail promoted the expression of P-gp, Bmi-1, Nanog, and Oct-4. Furthermore, P-gp, Bmi-1, Nanog, and Oct-4 induced by CCL21 were almost reversed after Snail knockdown by siRNA.

Different stimulation can upregulate Snail, but the underlying mechanisms are different. Either transcription of Snail or enhanced stability will lead to Snail accumulation [46]. Previous study has shown that Nodal, a member of TGF-*β* family, strengthens both the stability and transcription of Snail and induce EMT in cancer cells, while TNF-*α* enhances the stability, respectively [47, 48]. In our study, exposure to CCL21 could increase phosphorylation of GSK-3*β* at Ser9 residues and inhibit the activity of GSK-3*β*, leading to the stabilization of Snail. We further identify this suppose through detecting that ubiquitination of Snail was decreased after CCL21 treatment in HCT116 cells. Furthermore, GSK-3*β* binded with Snail at protein level was decreased, which may make a contribution to the less ubiquitination of Snail. The results of western blotting and immunofluorescence indicated that increased Snail was almost located in nucleus and promotes Snail to fulfill its roles as a transcription factor. In this study, it was indicated that PI3K/AKT signaling pathway is responsible for CCL21 inhibited GSK-3*β* activity and then Snail upregulation in HCT116 cells. Previous studies have mentioned that AKT/GSK-3*β* signaling pathway can modulate the stability of Snail [49, 50]. TGF-*β*/Smads, p38MAPK, and JAK/Stat3 are involved in regulating Snail expression [51–56]. Although CCL21 can trigger TGF-*β*/Smads, p38MAPK, and JAK/Stat3 signaling slightly in HCT116 cells, Snail level in the presence of specific inhibitors remains unaffected. We supposed that exposure to CCL21 can inhibit GSK-3*β* activity and change the levels of transcription factors, which was able to promote the transcription of Snail. However, this process needs more evidence to be explained.

## 5. Conclusions

In conclusion, we demonstrated that CCL21 is able to promote chemoresistance and cancer stem cell properties in HCT116 cells and Snail plays an important role during this process. We supposed a model in which CCL21 promotes the stabilization and transcription of Snail by triggering AKT activity and inhibiting GSK-3*β* activity. CCL21 could promote chemoresistance in cancer cell by its receptor CCR7; hence, CCR7 may be seen as a target in the future cancer therapy. In addition, the upregulation of CCL21 could be considered as an indicator of chemoresistance, which helps us to regulate the therapeutic schedule. Of course, these guesses need further validation.

## Abbreviations

P-gp: P-glycoprotein
CRC: Colorectal cancer
GPCRs: G protein-coupled receptors
CCR7: CC chemokines receptor 7
AML: Acute myeloid leukemia
MDR: Multidrug resistance
Dox: Doxorubicin
5-FU: 5-fluorouracil
bFGF: Basic fibroblast growth factor
Treg: Regulatory T cell.
